# N6-methyladenosine-related single-nucleotide polymorphism analyses identify oncogene RNFT2 in bladder cancer

**DOI:** 10.1186/s12935-022-02701-z

**Published:** 2022-10-05

**Authors:** Jiancheng Lv, Qiang Song, Kexin Bai, Jie Han, Hao Yu, Kai Li, Juntao Zhuang, Xiao Yang, Haiwei Yang, Qiang Lu

**Affiliations:** Department of Urology, The First Affiliated Hospital of Nanjing Medical University, No. 300 Guangzhou Road, Nanjing, 210029 China

**Keywords:** Bladder cancer, Single-nucleotide polymorphisms, N6-methyladenosine, Ring finger protein, Transmembrane 2, Proliferation, Migration

## Abstract

**Background:**

Single-nucleotide polymorphisms (SNPs) in N6-methyladenosine (m6A) related genetic locus play significant roles in tumorigenesis and development. The expression level of many oncogenes and tumour suppressor genes changed because of m6A-associated SNPs. In addition, the relationship between m6A-SNP and bladder cancer (BCa) has not been well studied.

**Methods:**

We screened m6A-SNPs in BCa by combining m6A-SNPs data and GWAS-SNPs data. Expression quantitative trait loci (eQTL) and differential expression gene (DEGs) analyses were performed. In ring finger protein, transmembrane 2 (RNFT2), rs3088107 (C  > G) was found to have significant eQTL signals and make RNFT2 gene differentially-regulated mostly in BCa. We validated the expression level of RNFT2 in 32 pairs of BCa tissues and eight BCa cell lines by quantitative real-time PCR (qRT-PCR). Functional assays were performed to investigate the role of rs3088107 and RNFT2 in BCa in *vitro*.

**Results:**

We identified 673 m6A-SNPs, which were associated with BCa. Of these m6A-SNPs, 221 showed eQTL signals, amongst which, rs3088107 in RNFT2 showed significant eQTL signals. Results of bioinformatic analyses showed that 11 genes with m6A-SNPs had a differential expression level in BCa. RNFT2 was predicted to be significantly up-regulated in BCa. The qRT-PCR results validated that RNFT2 was highly expressed in our own BCa tissues and cell lines. High expression of RNFT2 also indicated a worse overall survival. We also revealed that rs3088107 (C  > G) could inhibit the expression and m6A modification of RNFT2 by qRT-PCR, western-blot and m6A-RIP assays. Moreover, the results of functional assays indicated that RNFT2 promoted BCa cell proliferation and migration.

**Conclusion:**

This research found that m6A-SNPs were associated with oncogene RNFT2 in BCa. Furthermore, m6A-SNPs showed great application potential as a new BCa diagnostic biomarker and prognostic indicator.

**Supplementary Information:**

The online version contains supplementary material available at 10.1186/s12935-022-02701-z.

## Background

Bladder cancer (BCa) is considered as a prevalent cancer worldwide [1]. In recent years, the incidence and mortality of BCa increased rapidly worldwide [2, 3]. Based on the depth of invasion, BCa can be divided into non-muscle invasive bladder cancer (NMIBC) and muscle invasive bladder cancer (MIBC) [4]. More than 90% of patients with NMIBC have a higher 5-year survival rate than those with MIBC [5]. However, NMIBC patients tend to relapse frequently, particularly those with high-grade urothelial carcinoma [5]. At present, effective biomarkers in the diagnosis and treatment of BCa are still lacking.

Etiological research has revealed that genetic variations play a crucial role in BCa tumorigenesis and development [6, 7]. As an important genetic variation, single-nucleotide polymorphisms (SNPs) cannot be ignored during BCa development [5]. Considering the increasing number of in-depth research on genome-wide association studies (GWAS), numerous BCa-associated SNPs have been found [8]. In 2008, many SNPs were found to be related to BCa by GWAS. Amongst these, rs9642880 on chromosome 8q24, which was located near the c-Myc gene, showed the most significant relevance [9]. Based on previous studies, SNPs can affect gene expression in a number of ways [10–12]. In addition to the effects of amino acids, some changes in post-transcriptional modifications cannot be ignored [10–12].

N6-methyladenosine (m6A) modification, a methylation at the adenosine N6 position, is the abundant modification form in human [13]. RNA m6A modification is dynamically regulated by demethylases (writers) and methyltransferases (erasers) [14]. The m6A modification can be recognised by a set of RNA-binding proteins (readers) [14]. The metabolism, function and localisation of RNA can be influenced by m6A modifications [15]. By altering the expression and function of some oncogenes and tumour suppressor genes, m6A modifications can affect the occurrence and progression of multiple tumours [16–19]. Many cancer-associated signalling pathways are activated and inhibited by m6A modifications [16–19]. During the m6A modification process, the m6A regulators like METTL3/METTL14 proteins could recognize and methylate the DRACH motif (D = A, G, U; R = A, G; H = A, C, U) in RNA sequence, which we call m6A sites. However, when the base of DRACH motif has an SNP mutation, m6A regulators can’t recognize these motifs properly and the process of m6A modification will be affected [20, 21]. Therefore, m6A-SNPs are considered as a critical type of genetic variations, which can affect the development of many diseases, particularly cancers [22–24]. The identification of BCa associated with m6A-SNPs can provide comprehensive understanding of the underlying mechanism of BCa and help us to find effective diagnostic biomarkers and therapeutic targets.

In this research, we identified 673 SNPs by combining m6A-SNPs data and BCa GWAS-SNPs data. After expression quantitative trait loci (eQTL) were performed, rs3088107 (C  > G) in 3'-UTR region of Ring finger protein, transmembrane 2 (RNFT2) was found to have significant eQTL signals. The expression level of RNFT2 was found to be up-regulated in BCa by bioinformatic and experimental methods. And we also revealed that rs3088107 (C  > G) could decrease the expression and m6A modification of RNFT2 by bioinformatic analyses and experiments. In *vitro* functional assays validated that RNFT2 could promote BCa cell proliferation and migration.

## Materials and methods

### Identification of BCa-associated m6A-SNPs

We first searched for BCa-SNPs in the GWAS Catalog website (https://www.ebi.ac.uk/gwas/) to determine m6A-SNPs related to BCa. We downloaded the list of M6A-SNPs from the m6AVar database (http://m6avar.renlab.org/help.html) to identify M6A-SNPs amongst these SNPS [25]. By combining the GWAS summary data set with the m6A-SNPS list and setting *P* < 0.05 as the filtering condition, we found the m6A-SNPs associated with BCa.

### Expression quantitative trait loci analysis of BCa-associated m6A-SNPs

BCa-related m6A-SNPs may affect RNA modification; thus, they may take effect by regulating gene expression. We conducted a search of cis-eQTL analyses using public data through the HaploReg browser to find whether or not m6A-SNPs change the expression of local genes (https://pubs.broadinstitute.org/mammals/haploreg/haploreg.php). *P* < 0.05 was considered statistically significant.

### Differential expression analysis

The corresponding genes of the identified eQTL m6A-SNPs were further evaluated on the basis of the differential expression between the BCa patients and control group. Thus, the GSE166716 (n = 24) datasets from the GEO database (https://www.ncbi.nlm.nih.gov/geo/) and the TCGA datasets were downloaded and analysed using R software. In addition, immunohistochemical datasets were retrieved from The Human Protein Atlas (HPA) database (https://www.proteinatlas.org/).

### Patient samples and cell lines

A total of 32 pairs of BCa tissues used in this study were obtained from patients who underwent radical surgery between 2014 to 2019 at the First Affiliated Hospital of Nanjing Medical University. All human-associated tissues used in this research were approved by the Ethics Committee of The First Affiliated Hospital of Nanjing Medical University. The informed consent has been signed by all patients before their tissues were acquired. The BCa cell lines (5637, BIU87, RT4, UMUC3, J82 and T24) and normal urothelial cell line (SV-HUC) were collected from the Type Culture Collection of the Chinese Academy of Sciences (Shanghai, China).

### Immunohistochemistry

Paraffin-embedded tumors from BCa patients were sliced to 4 mm slides. The different grades of ethanol were used to rehydrate the tissue slides. Then we isolated the antigen by utilizing a microwave. The slides were dipped in 3% H_2_O_2_ and then incubated with RNFT2 antibody at 4 °C overnight. The slides were treated with the HRP-conjugated antibody. The results were observed with a microscope. The degree of positivity was identified by at least two pathologists according to the proportion of positive tumor cells.

### Cell culture and transfection

UMUC3 and T24 cells were cultured in DMEM medium (Gibco, USA) with 10% foetal bovine serum (BI, Israel). We maintained the cells in a 37 °C constant-temperature incubator containing 5% CO_2_.

Rs3088107 wild (C) or mutant (G) vectors and RNFT2 small-interfering RNAs (siRNAs) were purchased from GenePharma (GenePharma, Shanghai, China). The UMUC3 and T24 cells grown to 60% confluence in six-well plates were transfected with relative vectors or siRNAs by using the Lipofectamine 3000 kit (Invitrogen, USA).

### RNA isolation and quantitative real-time PCR (qRT-PCR)

RNAs were extracted from BCa tissues and cells by using a Trizol reagent (Invitrogen, USA). Then, RNAs were reversely transcripted into cDNAs using HiScript II (Vazyme, China). QRT-PCR experiments were applied using the StepOne Plus Real-Time PCR system (Applied Biosystems, USA). β-actin was used as internal control. The primers were purchased from TsingKe (TsingKe, Nanjing, China), which are listed in Additional file 1: Table S1.

### Protein extraction and Western-blot

We lysed the cells or tissues via using RIPA buffer (Sigma, USA). We quantified the concentrations of protein extractions by bicinchoninic acid (BCA) assays (Beyotime, China). Then we isolated and transferred the protein to polyvinylidene fluoride (PVDF) membrane (Millipore, USA) via SDS-PAGE. After blocked by using 5% skim milk, the PVDF membrane was incubated with primary (Protech, USA) and secondary antibodies (Protech, USA). Chemiluminescence (Bio-Rad, USA) and Image Lab Software were used to evaluate the expression level of proteins.

### Prediction of m6A modification

We predicted the m6A modification level of RNFT2 3'-UTR region with or without rs3088107 by using SRAMP database (http://www.cuilab.cn/sramp/). We imported the 3'-UTR sequence before and after rs3088107 occurrence into the database, which then could predict the m6A modification level based on the potential DRACH motif (D = A, G, U; R = A, G; H = A, C, U) in the sequence.

### M6A modifcation quantifcation of RNA

We measured the total RNA m6A modification level by using the epiQuik m6A RNA methylation quantifcation kit (Epigentek Group Inc, USA). Specifically speaking, we added 200 ng RNA into each well loaded with binding solution. After incubating for 90 min, we added capture antibody reagent, detection antibody reagent and enhancer reagent into all wells in turn. The absorbance was measured with 450 nm wavelength. The m6A content of total RNA were calculated with a standard curve.

### M6A RNA immunoprecipitation assay (MeRIP)

293 T cells which transfected with either the rs3088107 wild (C) vectors or mutant (G) vectors were treated with DNase I (Sigma Aldrich, USA). Then we fragmented RNAs by sonication for 15 s on an ice water mixture. Immunoprecipitations of RNAs fragments were applied via anti-m6A antibody (1:1000, Abcam, USA) which was previously treated with protein A/G beads (Life Technologies, USA) by using Magna RIP Kit (Millipore, MA). We then treated the samples with Proteinase K (20 mg/ml) at 42 °C for 1.5 h. After washing the samples several times, the RNAs were extracted with phenol: chloroform: isoamyl alcohol. The qRT-PCR was used to assess the level of RNFT2, which normalizing to input.

### Cell proliferation assay

We seeded 2000 UMUC3 or T24 cells in 96-well plates to investigate the role of RNFT2 in tumour proliferation. Cell viability was determined every 24 h (24, 48, 72 and 96 h) using the cell counting kit-8 (CCK-8) technique (Dojindo, Japan). The absorbance values at 450 nm were detected by a microplate reader (Tecan, Switzerland).

### Cloning formation

UMUC3 or T24 cells were seeded at 1000 cells per well in six-well plates. After 10 days, we fixed the cell colonies using 4% paraformaldehyde. Then, the fixed cell colonies were stained by 0.1% crystal violet. We calculated the cell colonies using Image J software.

### Scratch wound healing assay

After being grown over 90% confluence in six-well plates, the monolayer UMUC3 or T24 cells were scraped off by using a 200 μL pipette tip. Then, the cells were cultured in a serum-free medium. Cells were imaged using a microscope (Olympus, Japan) at 0 and 48 h. The migration rate was calculated using a formula based on the distance travelled by cells divided by the starting distance.

### Transwell assays

A total of 20,000 UMUC3 or T24 cells were seeded in the upper chamber (Corning, USA) of the transwell system with 200 µL of serum-free medium to investigate the migration ability. In the meantime, 600 µL of medium with 10% FBS was added into the lower chamber. The cells of top chambers were fixed with 4% paraformaldehyde for 15 min after 48 h. Then, we stained the cells for 30 min by using 0.5% crystal violet. We visualised and photographed the cells in the upper chamber using an Olympus microscope.

### Statistical analysis

SPSS version 22.0 (IBM Corp., Armonk, NY, USA) was used to analyse the data. The results were presented as mean ± standard deviation (means ± SD). We investigated the differences amongst different groups by using Student’s t-test and one-way ANOVA test. The Kaplan–Meier method was used to analyse the overall survival. When *P* values were less than 0.05, the results were considered statistically significant.

## Results

### BCa-associated m6A-SNPs

**Fig. 1:**
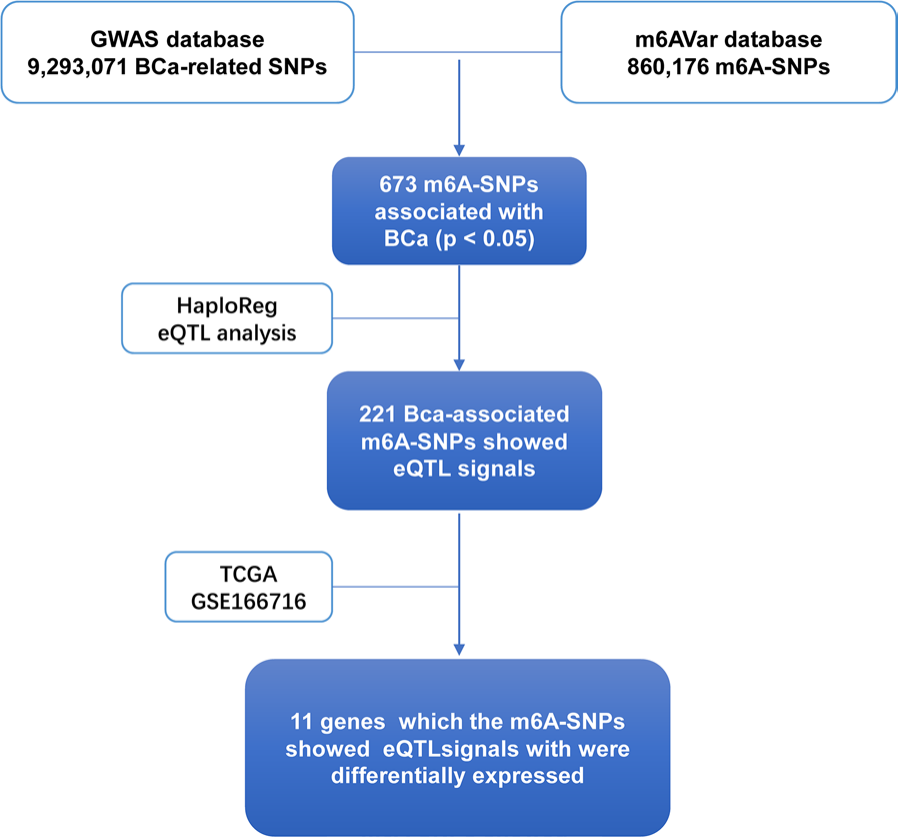
Flow diagram of study analysis and results

By taking the intersection of 9293071 BCa-SNPs downloaded from the GWAS Catalog website and 860176 m6A-SNPs downloaded from the m6AVar Database, a total of 673 BCa-associated m6A-SNPs were obtained (Fig. 1). The identities of the 9293071 BCa-SNPs and 860176 M6A-SNPs were listed in Additional file 2: Table S2.

### eQTL analysis

In the next step, we performed eQTL analysis on the 673 BCa-associated m6A-SNPs. The results showed that 221 m6A-SNPs had significant eQTL signals including rs3088107 (*P* < 0.05, Additional file 3: Table S3, Additional file 4: Figure S1A). In addition, we found 89 BCa-associated m6A-SNPs located in introns, 34 located in CDS, 31 located in 3'-UTR, 15 located in exons and four located in 5′-UTR (Additional file 4: Figure S1B). The top 20 m6A-SNPs with significant eQTL signals were shown in Table 1 by *p*-value ascending order, among which 17 m6A-SNPs led to a loss of m6A function, and three led to a gain of m6A function.

**Table 1:** The top 20 most significant m6A-SNP-eQTLs in BLCA

SNP rsID	CHR	Position	m6A_ID	Gene	Confidence-level	Gene_region	m6A-function	allele	eQTL	P value
rs3001721	chr1	43410117	RMVar_ID_1188900	SZT2	Low	Intron	Gain	G > C	2 hits	0.000151
rs7492724	chr14	37504161	RMVar_ID_976146	MIPOL1	Low	Intron	Loss	T > C	3 hits	0.000153
rs2251802	chr1	43451967	RMVar_ID_500132	HYI	High	CDS	Loss	A > G	2 hits	0.000175
rs6503807	chr17	47695542	RMVar_ID_1069575	TBKBP1	Low	Intron	Gain	T > A	3 hits	0.00019
rs815971	chr7	55422055	RMVar_ID_745791	LANCL2	High	Intron	Loss	G > A	19 hits	0.000206
rs2305209	chr10	71646775	RMVar_ID_883898	CDH23	Low	Intron	Gain	A > G	1 hit	0.000711
rs7739747	chr6	1.3E + 08	RMVar_ID_328198	SAMD3	High	Unknown	Loss	A > G	63 hits	0.000714
rs6533370	chr4	1.09E + 08	RMVar_ID_528592	RPL34-AS1	High	Intron	Loss	A > T	8 hits	0.000789
rs1053522	chr1	32894743	RMVar_ID_496190	TMEM54	Medium	Exon	Loss	C > G	1 hit	0.000997
rs9543517	chr13	74066194	RMVar_ID_190338	KLF12	High	Unknown	Loss	G > G	42 hits	0.001263
rs660118	chr11	65967705	RMVar_ID_163525	SART1	Medium	Unknown	Loss	T > C	6 hits	0.001275
rs758392	chr17	46940493	RMVar_ID_592763	GOSR2	Medium	Intron,3'UTR	Loss	T > C	1 hit	0.001285
rs1043550	chr7	1.29E + 08	RMVar_ID_248432	CALU	High	3'UTR	Loss	T > C	15 hits	0.00145
rs14157	chr11	66002307	RMVar_ID_163586	BANF1	Medium	5'UTR	Loss	G > C	20 hits	0.001502
rs3211270	chr5	58459874	RMVar_ID_441161	PLK2	Medium	CDS	Loss	T > A	1 hit	0.001695
rs17206904	chr6	31464727	RMVar_ID_362456	HCP5	Medium	Intron	Loss	T > G	88 hits	0.001815
rs56375210	chr16	81110095	RMVar_ID_1042255	U6	Low	Exon	Loss	T > C	1 hit	0.002085
rs3825175	chr12	1.22E + 08	RMVar_ID_542999	ORAI1	High	CDS	Loss	T > C	27 hits	0.002157
rs72824491	chr17	15502439	RMVar_ID_196207	CDRT4	High	Intron	Loss	C > G/T	1 hit	0.002173
rs6674599	chr1	200849850	RMVar_ID_368900	CAMSAP2	Medium	CDS	Loss	C > G	1 hit	0.0002318

### Differential expression analysis

**Fig. 2:**
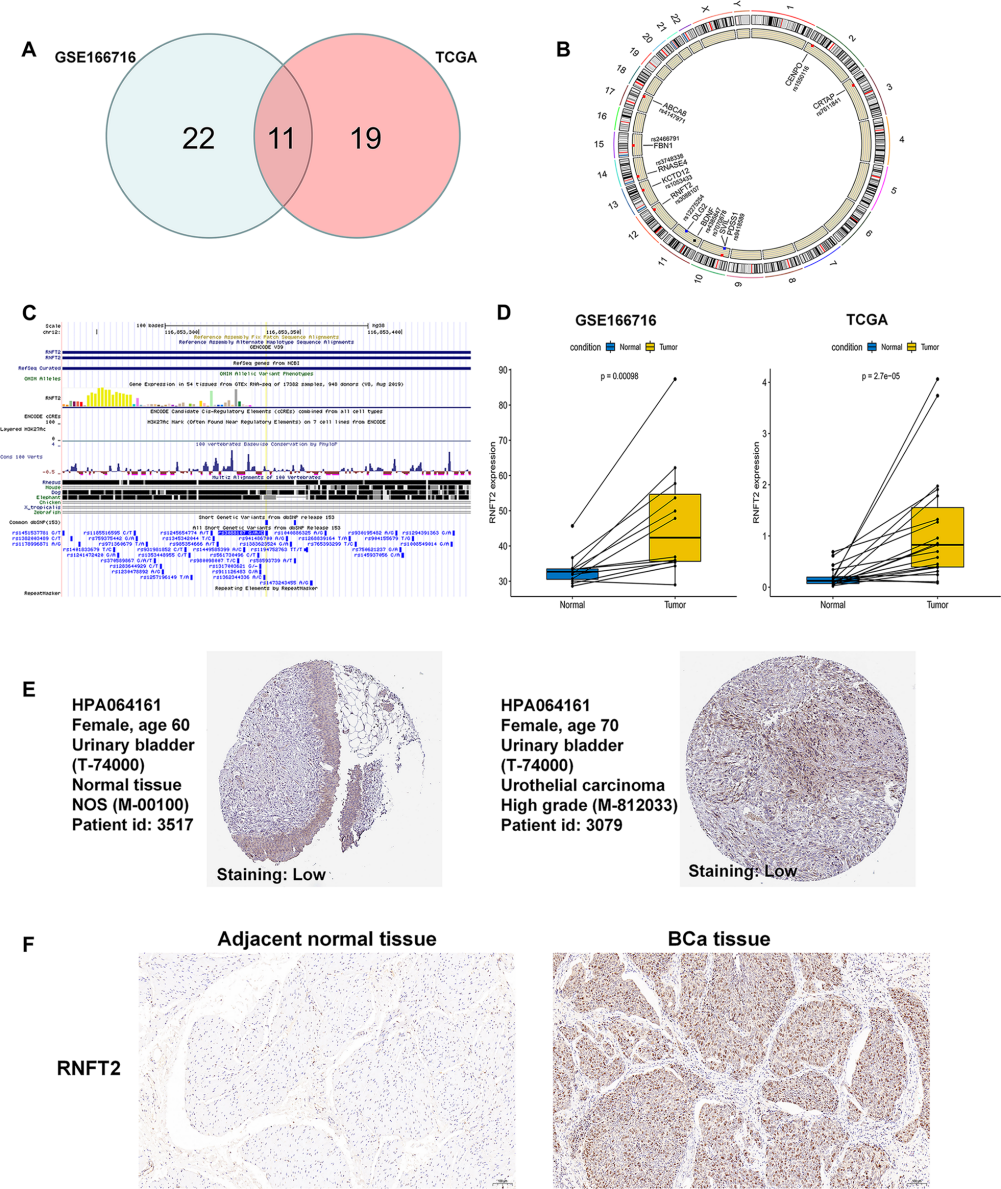
Regional association plots of the rs3088107 locus and the key gene identification. **A** The Venn plot of the 11 dysregulated m6A-SNP-eQTL genes. **B** Circus plots of chromosome distributions of the 11 dysregulated genes. **C** The linear plots of chromatin loops, states and signatures associated to rs3088107 using UCSC Genome Browser. **D** The expression of RNFT2 in BCa was higher than that in normal tissues from the TCGA BLCA and GEO datasets (GSE166716). **E** Validation of the expression of RNFT2 in BCa and normal tissues in the Human Protein Atlas (HPA) database. **F** Validation of the expression of RNFT2 in our own BCa tissues and adjacent normal tissues by IHC

The detailed positions and the local genes (the host genes where the SNPs occur) of the 221 BCa-m6A-SNPs were investigated by m6AVar database (Additional file 3: Table S3). We confirmed the expression levels of the local genes by integrating and analysing the TCGA BLCA (n = 19) and GSE166716 (n = 12) datasets. Eleven genes were dysregulated in both two datasets (Fig. 2A). Amongst the 11 dysregulated genes with identified eQTL m6A-SNPs, three genes were up-regulated in BCa tissues, whereas eight were down-regulated (Table 2). Ten of which had m6A function loss, whereas only one had m6A function gain (Table 2). Studies have shown that m6A modification affects the post-transcriptional modification and translation of genes by locating in the 3'-UTR region [19]. Of the three upregulated genes, RNFT2 showed this potential. The detailed position of rs3088107 in chromosome 12 was investigated. And the result confirmed that it was located in the 3'-UTR region of the RNFT2 gene (Fig. 2B and C). The expression of RNFT2 was found to be up-regulated in BCa tissues compared with normal tissues by analysing TCGA BLCA (n = 19) and GSE166716 (n = 12) datasets (Fig. 2D). The protein level of RNFT2 was confirmed to be up-regulated in BCa tissues compared with normal tissues by using the HPA database (Fig. 2E). And we also confirmed the expression level of RNFT2 in our own BCa samples by immunohistochemistry (IHC) method. The results showed RNFT2 was highly expressed in BCa tissues when compared with adjacent normal tissue (Fig. 2F).

**Table 2:** The dysregulated genes of the identified eQTL m6A-SNPs in BCa

SNP_rsID	CHR	Position	m6A_ID	Gene	DIFF	Gene_region	Confidence-leval	m6A-function	allele	P-value
rs3088107	chr12	116853335	RMVar_ID_466276	RNFT2	Up	3'UTR	High	Functional Loss	C > G	0.03746
rs9418589	chr10	26727174	RMVar_ID_552901	PDSS1	Up	Unknown	High	Functional Loss	G > C	0.04667
rs1550116	chr2	24799727	RMVar_ID_501862	CENPO	Up	exon	High	Functional Loss	G > A	0.01965
rs7611841	chr3	33114704	RMVar_ID_646207	CRTAP	Down	Unknown	High	Functional Loss	A > G	0.02687
rs4385847	chr11	27686182	RMVar_ID_554056	BDNF	Down	intron	Medium	Functional Loss	T > A	0.04668
rs4147971	chr17	68932091	RMVar_ID_1076900	ABCA8	Down	intron	Low	Functional Loss	A > G	0.04653
rs1053433	chr13	76881756	RMVar_ID_625520	KCTD12	Down	3'UTR	High	Functional Loss	C > T	0.009932
rs2466791	chr15	48450644	RMVar_ID_650541	FBN1	Down	intron	High	Functional Loss	A > T	0.002605
rs12275254	chr11	83632881	RMVar_ID_926147	DLG2	Down	intron	Low	Functional Loss	A > G	0.02632
rs7070678	chr10	29523675	RMVar_ID_878418	SVIL	Down	CDS	Low	Functional Gain	T > C	0.01269
rs3748338	chr14	20699417	RMVar_ID_682222	RNASE4	Down	CDS	Medium	Functional Loss	A > G	0.04457

### RNFT2 was up-regulated in BCa tissues and cell lines and associated with poor prognosis

**Fig. 3:**
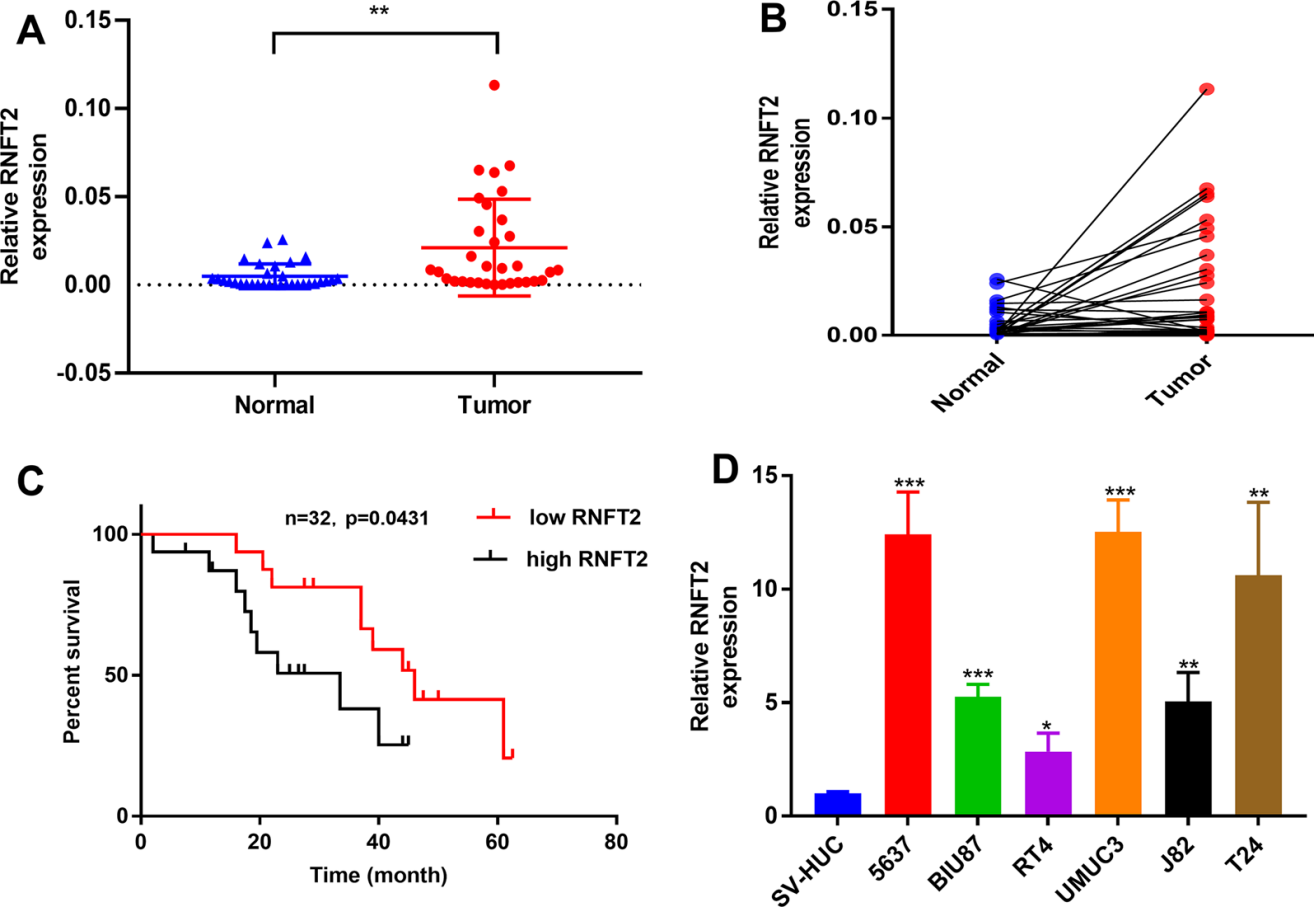
RNFT2 was up-regulated in BCa tissues and cell lines and positively associated with poor survival. **A** and **B** The expression levels of RNFT2 in 32 pairs of BCa tissues and adjacent normal tissues (**P < 0.01, Student’ s t-test). **C** Kaplan–Meier analysis showed the relation between RNFT2 expression and overall survival in 32 BCa patients. **D** The expression levels of RNFT2 in 7 BCa cell lines and the SV-HUC cell line (*P < 0.05, **P < 0.01, ***P < 0.001, ns = no significant, Student’s t-test). Data are mean ± SD, n = 3

We detected the expression level of RNFT2 in 32 pairs of BCa tissues. The expression of RNFT2 was significantly up-regulated in BCa tissues compared with adjacent normal tissues (Fig. 3A and B). Prognostic analysis was performed. And the high RNFT2 expression group showed poor overall survival (Fig. 3C). While there was no significant correlation between RNFT2 and other clinicopathological parameters like age, gender, tumor size, tumor node metastasis (TNM) stage, and Histological grade (Table 3). We also investigated the expression level of RNFT2 in BCa cell lines. We found that RNFT2 was up-regulated in six BCa cell lines when compared with normal urothelial cell line (SV-HUC) (Fig. 3D).

**Table 3:** Correlations between the expression of RNFT2 and clinicopathological features in BCa patients

Characteristics	Case	RNFT2		*P* value
		Low	High	
All cases	32	16	16	
Age(years)				1.000
< 65	14	7	7	
≥ 65	18	9	9	
Gender				0.2200
Male	24	10	14	
Female	8	6	2	
TNM stage				0.3944
pTa-pT1	7	5	2	
pT2-pT4	25	11	14	
Histological grade				0.6851
Low	8	5	3	
High	24	11	13	
Tumor size(cm)				1.000
< 3	11	5	6	
≥ 3	21	11	10	

^*^P < 0.05

### SNP rs3088107 inhibited the expression and m6A modification of RNFT2

**Fig. 4:**
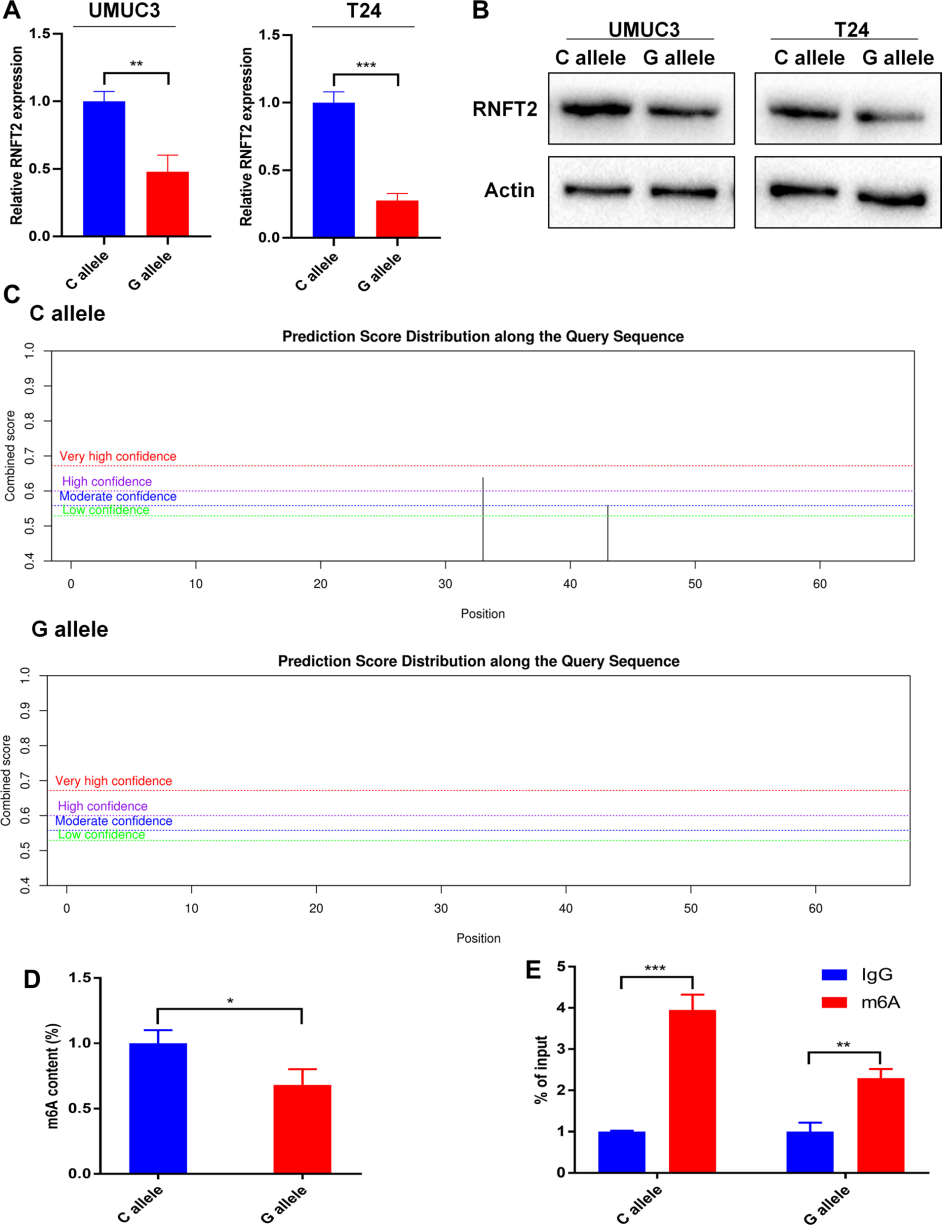
SNP rs3088107 attenuated the m6A modification and expression of RNFT2. **A** QRT-PCR results showed rs3088107 inhibited the expression of RNFT2 in UMUC3 and T24 cells (**P < 0.05, ***P < 0.001, Student’s t-test). **B** Western blot results showed rs3088107 inhibited the expression of RNFT2 in UMUC3 and T24 cells. **C** Results of SRAMP analyses showed m6A modification in RNFT2 3’-UTR was decreased after rs3088107 happened. **D** M6A modification quantification assays revealed that rs3088107 could decrease whole RNA m6A modification content. **E** Me-RIP assays indicated that rs3088107 could weaken the m6A modification of RNFT2 (**P < 0.05, ***P < 0.001, Student’s t-test). Data are mean ± SD, n = 3

The occurrence rate of rs3088107 in 15 BCa samples were investigated by Sanger sequencing. Among them, 9 samples were C allele and 6 samples were G allele. Due to the limited sample size, the effect of rs3088107 on the expression of RNFT2 in BCa tissues and the prognosis of BCa patients did not show a significant trend. However, the mean expression of RNFT2 in samples with C Allele was higher than that in samples with G allele (Additional file 5: Figure S2A). The mean survival of patients with C allele was worse than that of patients with G allele (Additional file 5: Figure S2B). There was no significant correlation between rs3088107 and other clinicopathological parameters like age, gender, tumor size, TNM stage, and Histological grade (Table 4). We transfected the wild rs3088107 vectors (C) and mutant rs3088107 vectors (G) into the UMUC3 and T24 cells. After 48 h, we validated the expression of RNFT2 via qRT-PCR and western-blot. The results showed that UMUC3 and T24 cells transfected with wild vectors (C) had higher expression level of RNFT2 compared with cells transfected with mutant vectors (G) (Fig. 4A-B). Through analyzing by SRAMP, we found the m6A modification of RNFT2 3'-UTR region was decreased because of SNP rs3088107 (C  > G) (Fig. 4C). Then we transfected the wild rs3088107 vectors (C) and mutant rs3088107 vectors (G) into the 293 T cells in order to perform the RNA m6A modification quantification assay and meRIP assay. The result of the m6A modification quantification assay showed that SNP rs3088107 (C  > G) decreased the whole m6A modification level of RNA (Fig. 4D). And the result of meRIP indicated that SNP rs3088107 could inhibit the m6A modification of RNFT2 (Fig. 4E).

**Table 4:** Correlations between the SNP rs3088107 and clinicopathological features in BCa patients

Characteristics	Case	Rs3088107		*P* value
		C	G	
All cases	15	9	6	
Age(years)				0.6670
< 65	6	4	2	
≥ 65	9	5	4	
Gender				0.4745
Male	11	6	5	
Female	4	3	1	
TNM stage				0.7921
pTa-pT1	3	2	1	
pT2-pT4	12	7	5	
Histological grade				0.2148
Low	2	2	0	
High	13	7	6	
Tumor size(cm)				0.7921
< 3	3	2	1	
≥ 3	12	7	5	

^*^P < 0.05

### SNP rs3088107 inhibited the proliferation and migration of BCa cells in vitro

**Fig. 5:**
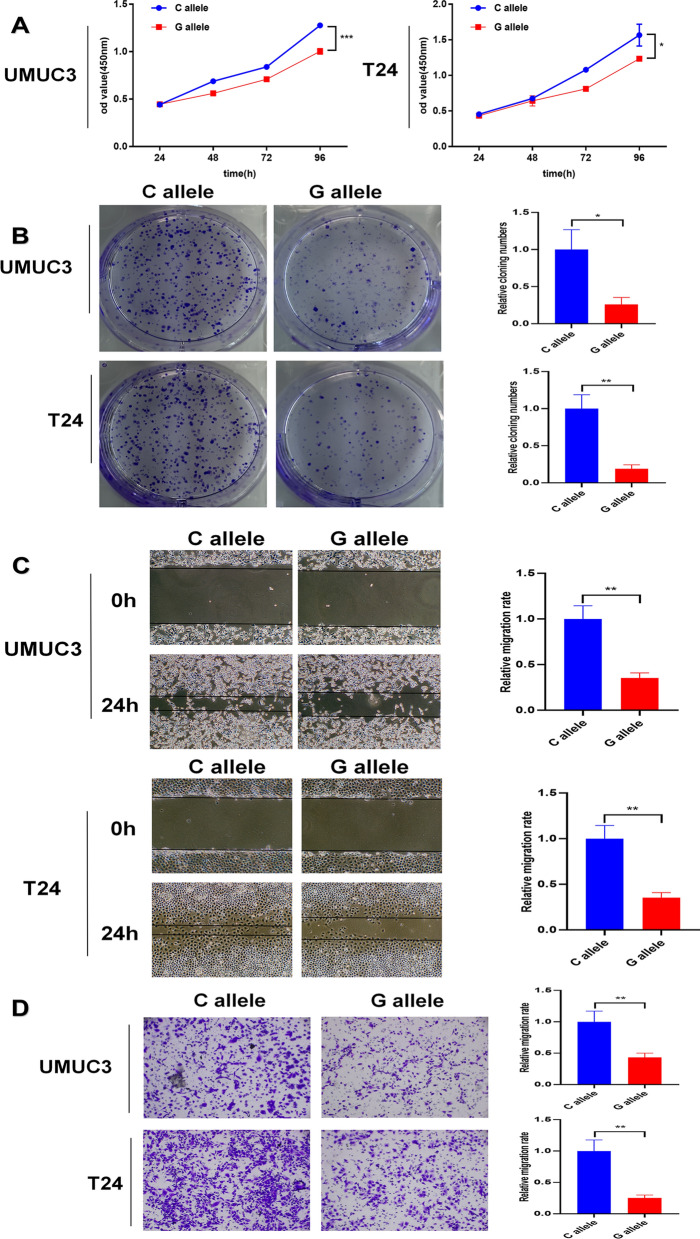
Rs3088107 inhibited the progression of UMUC3 and T24 cells in vitro*.* **A** CCK8 assay showed the rs3088107 mutant (G allele) could inhibit UMUC3 cells proliferation (*P < 0.05, ***P < 0.001, Student’s t-test). **B** Colony formation assay showed the rs3088107 mutant (G allele) could inhibit UMUC3 and T24 cells colony formation (*P < 0.05, **P < 0.01, Student’s t-test). **C** Scratch wound healing assays indicated that the rs3088107 mutant (G allele) could inhibit the migration rate of UMUC3 and T24 cells (**P < 0.01, Student’s t-test). **D** Transwell assays revealed that the rs3088107 mutant (G allele) could inhibit the migration rate of UMUC3 and T24 cells (**P < 0.01, Student’s t-test). Data are mean ± SD, n = 3

We transfected the wild rs3088107 vectors (C) and mutant rs3088107 vectors (G) into the UMUC3 and T24 cells. The results of CCK-8 assays revealed that the rs3088107 mutant (G allele) inhibit the proliferation ability of UMUC3 and T24 cells (Fig. 5A). Cloning formation experiments also indicated that rs3088107 mutant (G allele) could decrease the colony numbers of UMUC3 and T24 cells (Fig. 5B). In addition, we performed scratch wound healing assays and transwell assays. The results revealed that the rs3088107 mutant (G allele) could inhibit the migration rate of UMUC3 and T24cells (Fig. 5C and D).

### RNFT2 promoted the proliferation and migration of BCa cells in vitro

**Fig. 6:**
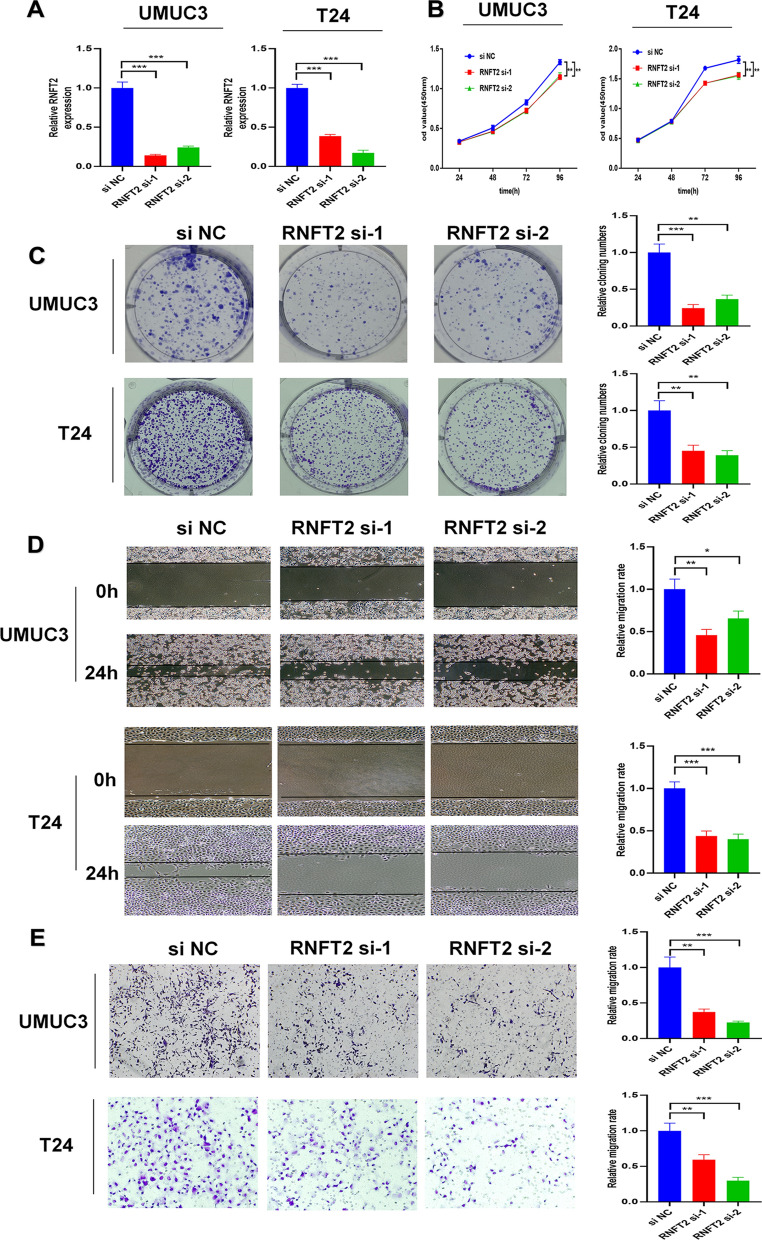
RNFT2 promoted the progression of UMUC3 and T24 cells in vitro. **A** The efficiency of RNFT2 si RNA transfection in UMUC3 and T24 cells (***P < 0.001, Student’s t-test). **B** CCK8 assay showed RNFT2 knockdown could inhibit UMUC3 cells proliferation (**P < 0.05, Student’s t-test). **C** Colony formation assay showed RNFT2 knockdown could inhibit UMUC3 and T24 cells colony formation (**P < 0.05, ***P < 0.001, Student’s t-test). **D** Scratch wound healing assays indicated that RNFT2 knockdown could inhibit the migration rate of UMUC3 and T24 cells (*P < 0.05, **P < 0.01, ***P < 0.001, Student’s t-test). **E** Transwell assays revealed that RNFT2 knockdown could inhibit the migration rate of UMUC3 and T24 cells (**P < 0.01, ***P < 0.001, Student’s t-test). Data are mean ± SD, n = 3

We transfected RNFT2 siRNAs into UMUC3 and T24 cells. The transfection efficiency was verified by qRT-PCR (Fig. 6A). We conducted functional assays to study the role of RNFT2 in vitro. The results of CCK-8 assays revealed that the knockdown of RNFT2 could inhibit the proliferation ability of UMUC3 and T24 cells (Fig. 6B). Cloning formation experiments also indicated that RNFT2 knockdown could decrease the colony numbers of UMUC3 and T24 cells (Fig. 6C). In addition, we performed scratch wound healing assays and transwell assays. The results revealed that the knockdown of RNFT2 could inhibit the migration rate of UMUC3 and T24cells (Fig. 6D and E).

## Discussion

BCa is a common cancer in the urinary system, which is also considered as the most common malignancy worldwide [26]. About 573,000 new cases were diagnosed with BCa, and 213,000 cases died of BCa every year [27]. The occurrence and progression of BCa are a process of multi-mutation [28]. At present, no suitable target has been found to guide the diagnosis and treatment of BCa. Given its high incidence and complex aetiology, finding effective diagnostic and therapeutic biomarkers is necessary.

RNA modifications have been reported as targets of many cancer-related cascades, which play a crucial role in regulating gene expression [16–19]. This process may result in tumorigenesis and metastasis [22–24]. M6A is an important and dominant process in RNA modifications [13]. M6A modification regulates molecular functions by mediating multiple steps of RNA processing such as RNA maturation, nuclear transport, RNA stability, splicing, translation and protein binding [29]. In addition, many m6A modifications on tumour-related genes directly affect tumour development and drug resistance [29]. Therefore, mutations occurring at the DRACH motif (D = A, G, U; R = A, G; H = A, C, U) may affect the entire modification and further interfere with the expression of some important genes [30]. As a common form of genetic mutation, SNPs may occur at any region of genes [31]. m6A-SNPs could occur in sites close to the methylation regions. It can also occur directly in the m6A modification sites. If m6A methylation was disturbed by SNP mutations, then many biological functions and pathways would be influenced [32]. Many m6A-SNPs, which were missense mutations, could intervene the transcription process. Some m6A-SNPs appear in UTRs or regions near stop codons, which can affect the binding of transcriptional regulators and RNA-binding proteins. Consequently, the stability and nuclear transport will be affected by this process. Moreover, m6A-SNPs could change the amino acid sequence and alter the secondary structure of proteins [33]. Therefore, m6A-SNPs are associated with many diseases, including cancers. Based on previous report, m6A-SNPs could inhibit the expression of SOD2 through binding hnRNPC [23]. However, as a multi-gene mutated disease, several m6A-SNPs remain unclear in BCa.

In this study, we screened out BCa-associated m6A-SNPs by integrating BLCA GWAS SNPs data and m6Avar data. In addition, a total of 673 m6A-SNPs was acquired, which were associated with BCa (*P* < 0.05). Further eQTL analysis was performed to screen for m6A-SNPs, which could influence the expression levels of local genes. A total of 221 BCa-associated m6A-SNPs showed significant eQTL signals. Amongst which, rs3088107 in Ring finger protein, transmembrane 2 (RNFT2) had significant eQTL signals, which means it can influence the expression of RNFT2. In order to figure out how rs3088107 regulated RNFT2, we performed necessary experiments. The results confirmed rs3088107 can attenuate the m6A modification and expression of RNFT2. The decreased expression of RNFT2 may be caused by m6A modification of 3'-UTR which could decrease the stability of mRNA induced by rs3088107. We confirmed the high expression level of RNFT2 in BCa tissues and cell lines. Further functional assays also indicated that RNFT2 could promote BCa cell proliferation and migration, while rs3088107 inhibited the proliferation and migration of BCa. So as a tumor suppressor SNP rs3088107 and an oncogene RNFT2 were identified. RNFT2, which is located on chromosome 12, encodes a protein formed with 444 amino acids. As a member of the ring finger family, RNFT2 was reported to participate in proteolytic process [34]. Research on RNFT2 was limited at least for now. It has been revealed that RNFT2 could inhibit inflammation response via influencing ubiquitination of IL-3Ra [34]. In the oncology field, the expression of RNFT2 in gastric cancer tissues was found positively associated with poor prognosis and high recurrence rates [35]. While in BCa, the function of RNFT2 had not been studied before.

## Conclusions

We demonstrated the role of RNFT2 as an oncogene in BCa for the first time. We confirmed that SNP rs3088107 (C  > G) could interfere the m6A modification in RNFT2 which could furtherly inhibit the expression of it. Therefore, RNFT2, which has a functional m6A-SNP, shows significant application potential as a diagnostic and therapeutic target in BCa.

## Supplementary Information

**Additional file 1: Table S1.** All PCR primers used in this research.

**Additional file 2: Table S2.** The identities of the 9293071 BCa-SNPs and 860176 M6A-SNPs.

**Additional file 3: Table S3.** The host genes where the 221 BCa-m6A-SNPs located in.

**Additional file 4: Figure S1.** Genome-wide analysis for the association between m6A-SNP and BCa. A The Manhattan plot shows –log10 p values of BCa associated m6A-SNPs. B BCa associated m6A-SNPs showed eQTL signals displaying a unique distribution pattern.

**Additional file 5: Figure S2.** The occurrence rate of rs3088107 and correlation with overall survival of BCa patients. A The occurrence rate of rs3088107 in 15 BCa patients. B Kaplan-Meier analysis showed the relation between rs3088107 and overall survival in 15 BCa patients.

## Data Availability

The datasets supporting the conclusions of this article are included within the article and its additional files.

## Abbreviations

SNP: Single-nucleotide polymorphisms
m6A: N6-methyladenosine
BCa: Bladder cancer
eQTL: Expression quantitative trait loci
DEG: Differential expression gene
RNFT2: Ring finger protein, transmembrane 2
qRT-PCR: Quantitative real-time PCR
NMIBC: Non-muscle invasive bladder cancer
MIBC: Muscle invasive bladder cancer
GWAS: Genome-wide association studies
