# Supplementary material for: N6-methyladenosine-related single-nucleotide polymorphism analyses identify oncogene RNFT2 in bladder cancer
Source: Cancer Cell Int. 2022 Oct 5;22:301. doi: 10.1186/s12935-022-02701-z (PMC9535860; doi:10.1186/s12935-022-02701-z)
Supplement: Supplementary file 1 — Additional file 1: Table S1. All PCR primers used in this research. [file 12935_2022_2701_MOESM1_ESM.docx]

**Table S1:** All PCR primers used in this research

| **Primers** |  | **sequence （5’-3’）** |
| --- | --- | --- |
| RNFT2 | Forward  Reverse | 5’-CACAGCAGCAACACGGATAAC-3’  5’-CTGCCTGATAAGCCCGAGA-3’ |
| β-Actin | Forward  Reverse | 5’-CTCCATCCTGGCCTCGCTGT-3’  5’-GCTGTCACCTTCACCGTTCC-3’ |
