# Supplementary figures and images for: N6-methyladenosine-related single-nucleotide polymorphism analyses identify oncogene RNFT2 in bladder cancer
Source: Cancer Cell Int. 2022 Oct 5;22:301. doi: 10.1186/s12935-022-02701-z (PMC9535860; doi:10.1186/s12935-022-02701-z)

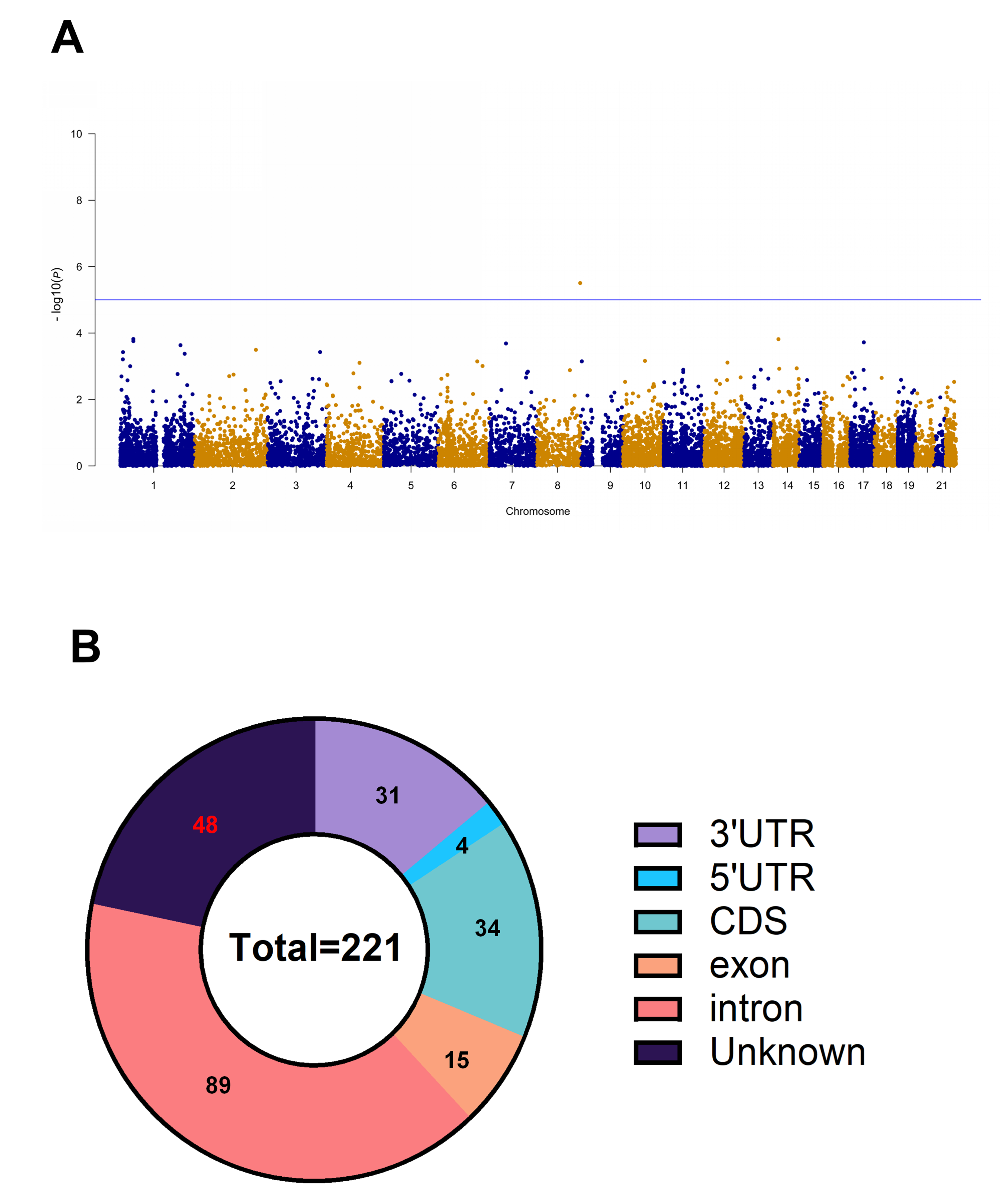

Supplement: Supplementary file 4 — Additional file 4: Figure S1. Genome-wide analysis for the association between m6A-SNP and BCa. A The Manhattan plot shows –log10 p values of BCa associated m6A-SNPs. B BCa associated m6A-SNPs showed eQTL signals displaying a unique distribution pattern. [file 12935_2022_2701_MOESM4_ESM.tif]

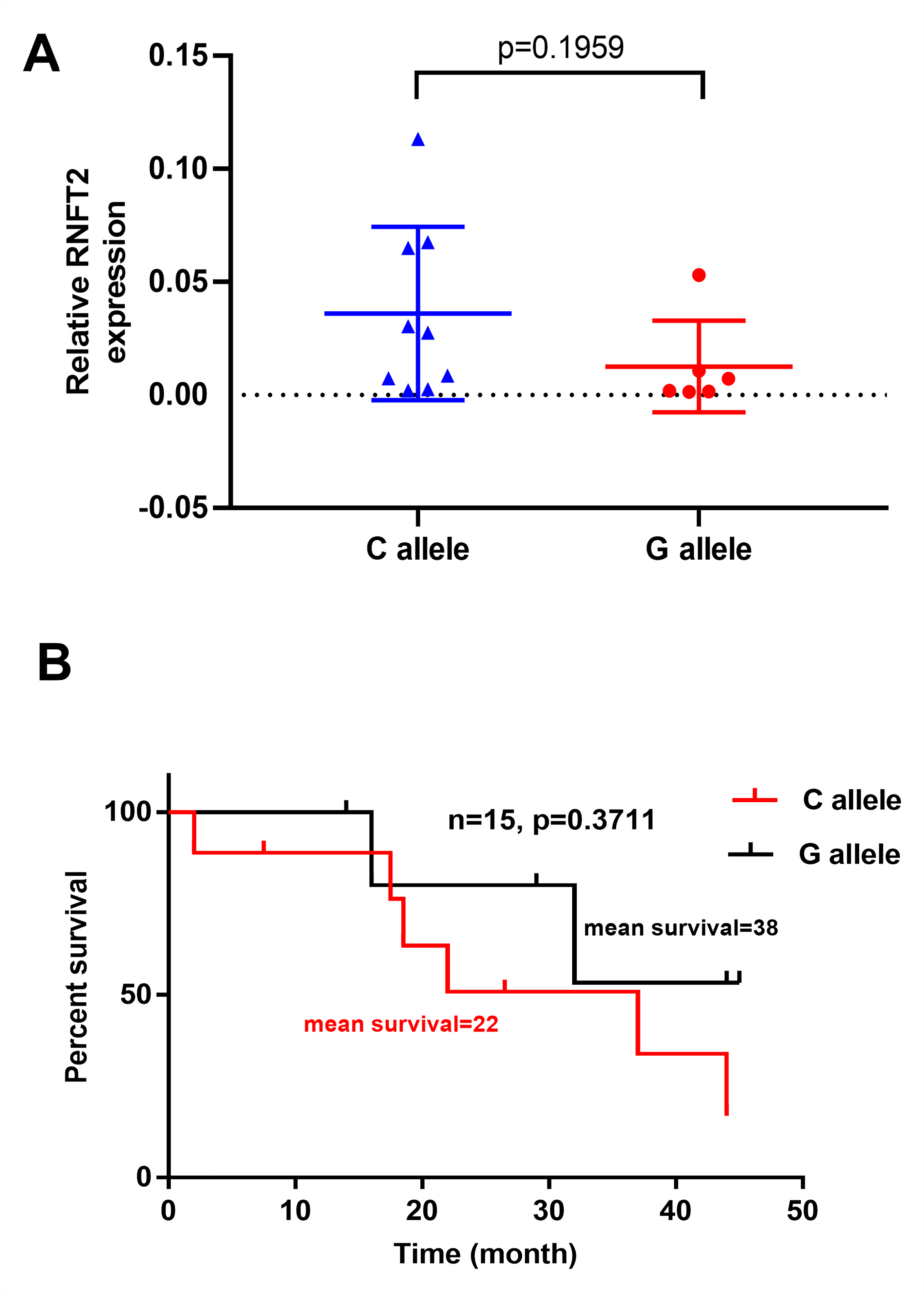

Supplement: Supplementary file 5 — Additional file 5: Figure S2. The occurrence rate of rs3088107 and correlation with overall survival of BCa patients. A The occurrence rate of rs3088107 in 15 BCa patients. B Kaplan-Meier analysis showed the relation between rs3088107 and overall survival in 15 BCa patients. [file 12935_2022_2701_MOESM5_ESM.tif]
